# Sleep Problems and Workplace Violence: A Systematic Review and Meta-Analysis

**DOI:** 10.3389/fnins.2019.00997

**Published:** 2019-10-01

**Authors:** Nicola Magnavita, Enrico Di Stasio, Ilaria Capitanelli, Erika Alessandra Lops, Francesco Chirico, Sergio Garbarino

**Affiliations:** ^1^Post-graduate School in Occupational Health, Università Cattolica del Sacro Cuore, Rome, Italy; ^2^Department of Women/Child & Public Health, Gemelli General Hospital Foundation IRCCS, Rome, Italy; ^3^Department of Neuroscience, Rehabilitation, Ophthalmology, Genetics, Mother and Child Health (DINOGMI), University of Genoa, Genoa, Italy

**Keywords:** workplace violence, sleep quality, sleep problems, psychological trauma, neurophysiology, sleep disorders, bullying, stress

## Abstract

**Background:** This systematic review with meta-analysis was carried out to study the relationship between workplace violence and sleep problems.

**Methods:** The PRISMA statement was used to conduct a systematic search of the literature on PubMed/MEDLINE, Scopus, Sociological abstract, DOAJ, Web of Science, and Google Scholar databases. Of the original number of 749 studies, 34 were included in the systematic review, and 7 in the meta-analysis.

**Results:** A total of 119,361 participants from 15 different countries took part in these studies which were published between 1999 and 2019. Significant heterogeneity was observed among the studies (*I*^2^ = 96%). In a random-effects meta-analysis model, pooled odds ratio (OR) analysis revealed that there was a direct relationship between occupational exposure to violence and sleep problems (OR = 2.55; 95% CI = 1.77–3.66). According to the GRADE guidelines, the quality of evidence of the association was low.

**Conclusions:** The findings of this study demonstrate that occupational exposure to physical, verbal, or sexual violence is associated with sleep problems. Further research on the relationship between violence and sleep is needed so that appropriate measures can be taken to prevent violence and improve sleep hygiene in the workplace.

**Trial Registration Number:** PROSPERO International prospective register of systematic reviews (CRD42019124903) February 9, 2019.

## Introduction

### Rationale

Sleep is very important for workers' health, safety, well-being, and productivity (Garbarino et al., 2016c; Magnavita and Garbarino, 2017). Sleep loss can have serious detrimental effects on cognitive performance, including vigilant attention (Hudson et al., 2019), dexterity (Banfi et al., 2019), executive functioning and performance (Massar et al., 2019), and memory and emotional function (Cousins and Fernández, 2019). These may increase the rate of occupational road accidents, near-miss accidents (Garbarino et al., 2016a, 2017), and occupational injuries (Garbarino et al., 2016b), and lead to negative organizational and individual outcomes in the workplace. Considering insufficient sleep duration only, it is estimated that on an annual basis, the U.S. loses an equivalent of about 9.9 million working hours. The annual economic loss ranges between $280 billion and $411 billion (Hafner et al., 2017). The costs that result from poor quality of sleep are difficult to determine but very relevant (Garbarino et al., 2016c). Poor quality sleep may also impair occupational memory capacity (Xie et al., 2019) and productivity (Park et al., 2018), and may contribute to reduced control over emotions and aggression, thereby incrementing hostile and aggressive behavior at the workplace (Garbarino and Sannita, 2017). Aggression is considered to be a key component of social behavior that must be properly controlled (De Almeida et al., 2015). Although aggressions may be especially distressing for exposed workers (López-López et al., 2019), and distress is associated with a range of negative outcomes, including sleep problems (Vleeshouwers et al., 2015), previous research has mainly focused on examining the impact of job stress on sleep (Yang et al., 2018) or the relationship between work and sleep (Litwiller et al., 2017), generally without considering violence. The relationship between workplace violence and sleep is extremely important (Magnavita and Garbarino, 2017) since the workplace is the environment where individuals spend most of their time.

Violence is one of the most pervasive and poorly controlled problems in the workplace (Hart and Heybrock, 2017). Night and shift workers are among the most exposed (Fischer et al., 2019). Currently, there is no single, consistent definition of workplace violence (WV). Different types of physical and verbal abuse are grouped under the WV heading, and the perception of what constitutes violence varies according to different contexts and cultures (Magnavita, 2011). According to NIOSH/CDC [The National Institute for Occupational Safety Health (NIOSH) Centers for Disease Control Prevention (CDC) Workplace Violence Prevention for Nurses, 2013], WV can be classified into four types: Type 1, Criminal Intent, when no legitimate relationship exists between the perpetrator and the business or its employees and the perpetrator commits a crime (robbery, shoplifting, trespassing) in addition to violence; Type 2, Violence toward the worker on the part of the customer/client; Type 3, worker-on-worker, commonly referred to as lateral or horizontal violence; Type 4, personal relationship, in which a relationship between the perpetrator and the worker outside of work is transferred to the work environment. The first type of violence, which can often be fatal, affects mainly police officers, bank employees, taxi drivers, and traders. Type 2 is common in health care and teaching. Type 3 and 4 are possible in all types of work [The National Institute for Occupational Safety Health (NIOSH) Centers for Disease Control Prevention (CDC) Workplace Violence Prevention for Nurses, 2013]. WV can have a significant effect on workers' health. While physical injuries are immediately apparent, damage to the psychic and social spheres is more difficult to perceive. Of all the effects, impairment of the quantity and quality of sleep is probably the least frequently investigated consequence.

Differing definitions of the term “WV” make it impossible to obtain an accurate assessment of the extent of the phenomenon. Data from the 6th European Working Conditions Survey (EWCS), conducted in 35 countries in 2015, showed that 12 and 2% of European workers had been exposed to verbal abuse and physical violence, respectively, in the month prior to the study. A significant number of European workers also complained of difficulty in falling asleep (12% at least several times a week); others reported waking up repeatedly (17%) and waking up feeling tired (14%; Eurofound Sixth European Working Conditions Survey, 2017). However, the Eurofound researchers failed to investigate a possible association between the two phenomena. Minor psychological trauma experienced in the workplace can nevertheless be associated with sleep complaints (Magnavita, 2015; Magnavita et al., 2015). Bullying and other psychosocial work factors may also have an impact on sleep disturbances (Ansoleaga et al., 2015; Linton et al., 2015; van Geel et al., 2016). However, few studies have focused specifically on the relationship between bullying or workplace violence and sleep problems.

### Objectives

The aim of this review was to systematically review studies on the association between workplace violence (WV) and sleep problems (SPs), and to evaluate the prevalence of the latter in workers exposed to violence. Other objectives were to calculate the odds ratio for meta-analysis and to identify in published studies the occupational factors affecting the association between violence and sleep problems so that preventive measures could be taken.

### Research Question

The questions posed by this research are: Is occupational violence associated with sleep problems? Which neurophysiological mechanisms can explain this type of association?

## Methods

### Study Design

Systematic review and meta-analysis.

### Participants, Interventions, Comparators, Outcome (PICO)

P: workers. I: exposure to workplace violence. C: workers not experiencing violence at work. O: sleep problems.

### Systematic Review Protocol

The study protocol of this research was registered on PROSPERO, on February 9, 2019, with the following registration number: CRD42019124903.

### Search Strategy

A systematic search of the literature was carried out, between March and April 2019, on PubMed/MEDLINE, Scopus, Sociological abstract, DOAJ, Web of Science, and Google Scholar databases in accordance with the “Preferred Reporting Items for Systematic reviews and Meta-Analyses” (PRISMA) guidelines. To define the terms PICO, according to evidence based practice (Rathbone et al., 2017; FGCU Library, 2019), our search initially started by using only one or two PICO elements in combination. Once few relevant studies were found, the database's bibliographic references were examined and the search was refined using subject headings or keywords for searching for similar relevant articles. A comparison of the keywords chosen for this review with those used by studies on similar topics (Nielsen et al., 2018) was carried out. Keyword checking was performed by five authors independently, without the help of external experts. The electronic search strategy for PubMed used keywords related to the topic under investigation (workplace violence OR workplace bullying OR workplace sexual harassment) in conjunction with sleep quality or quantity and their synonyms (sleep OR sleep quality OR sleep quantity), properly combined by Boolean operators. The PICOS was adapted to the other databases. Only original studies with English abstracts and keywords, with a text written both in English and Italian were retrieved, with no limits of years considered. Data from gray literature were not included. Although review studies and commentaries were excluded from the present review, additional eligible studies were included after a hand-search of their reference lists.

### Data Sources, Studies Sections, and Data Extraction

The principal criterion for eligibility was the presence of occupational violence and sleep problems. All studies that took into consideration any type of violence at work or that indicated the presence of any type of sleep problem were included, while studies on WV that failed to provide any information on sleep were excluded. Similarly, studies that reported violence, bullying, or harassment of a non-occupational type (e.g., war violence, disasters, and non-occupational accidents) were excluded. Moreover, studies on violence and bullying in young people (e.g., bullying at school) or in family, and studies on epigenetic determinants of violence were excluded. All the papers that mentioned sleep problems among the observed effects of WV were included. Both quantitative and semi-quantitative studies with cross-sectional, retrospective, case-control, and prospective design were screened for inclusion. Second level studies (review studies) were excluded, although they were examined in order to identify further research to be included in this review.

After independently reviewing all titles/abstracts to identify potentially relevant articles, two authors (NM and FC) used the aforementioned inclusion/exclusion criteria to select studies on the basis of a full-text review. Disagreements were resolved by discussion with a third author (SG), who acted as the final referee. The selected studies that met the pre-defined inclusion/exclusion criteria and were related to the topic of interest were included in our systematic review. These studies were then examined to ensure that they fulfilled specific meta-analysis criteria. Papers that failed to provide sufficient data for the calculation of odds ratios with 95% confidence intervals were excluded. The authors computed the correct parameters for a number of articles.

**Figure 1:**
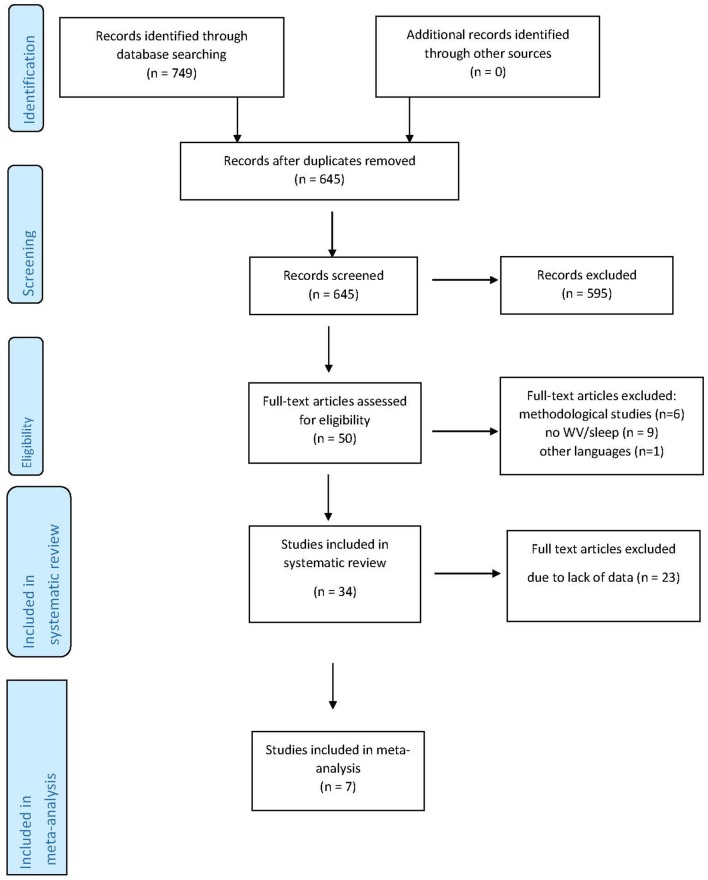
Article selection algorithm (PRISMA 2009).

Data concerning the country of study, job type, the type of WV, the method of measurement and the recall period, the type of sleep problem and, when applicable, the WV and sleep problem prevalence rate were extracted from each study. The relative risk (RR) or the odds ratio (OR) were also extracted when available. The authors carried out the data extraction process independently. The results of the studies were analyzed qualitatively, and when possible, also quantitatively for meta-analysis. The findings obtained were discussed by all the authors. Figure 1 illustrates the paper extraction flow diagram for this systematic review and meta-analysis.

### Study Quality Assessment

The quality of cohort and case-control studies was assessed by the Newcastle-Ottawa Scale (NOS) that evaluates selection, comparability and exposure criteria, attributing a maximum score of 9 points (Deeks et al., 2003; Wells et al., 2012). The quality of other studies was assessed using an adapted version of the Newcastle-Ottawa Quality Assessment Scale (NOS-A) for Case-Control/Cross-sectional studies (Modesti et al., 2016) that awards a maximum score of 10 points.

### Quantitative Data Synthesis

A recent systematic review of studies on workplace bullying concluded that research methods were too heterogeneous to enable meta-analysis to be performed (Linton et al., 2015). Bearing in mind this limitation and the considerable heterogeneity (different types of violence at work and various sleep problems) of the studies retrieved, an attempt was made to carry out a meta-analysis of the latter.

Statistical analysis on the relationships between WV and sleep problems based on odds ratios was used for the meta-analysis. Due to divergence in study designs, methods of data collection, and adjustment of the results, findings for this meta-analysis were reported according to a random-effects model (Clarke et al., 2010). The consistency of the results was tested by the heterogeneity indicator, I-squared (*I*^2^) statistic, with *I*^2^ of 25, 50, and 75% corresponding to a small, medium, and large degree of heterogeneity, respectively (Borenstein et al., 2011). Furthermore, the publication bias of the five effect sizes was tested by visual inspection, according to which an asymmetric shape in the funnel plots implied the existence of publication bias (Duval and Tweedie, 2000). The quality of evidence was assessed, with reference to the GRADE guidelines (Balshem et al., 2011). Analyses were performed with Review Manager 5.3 (RevMan5), Cochrane Community.

## Results

Research on databases resulted in a total of 749 studies. After the removal of duplicates and studies that failed to meet the eligibility criteria, 50 full-text articles were assessed. Three studies that failed to report sleep problems among the outcomes and five studies that failed to consider violence at work as a predictor were excluded, as was a study written in Icelandic. Six studies were excluded on the grounds that they were literature reviews or methodological articles, and a further study failed to meet the criteria as it was not conducted on workers. The final sample for systematic review consisted of 34 studies. Seven studies were included in the quantitative analysis since they contained prevalence data for calculating the odds ratio (Figure 1).

### Study Selection and Characteristics

The studies on workplace violence and sleep problems were mainly descriptive, cross-sectional, or retrospective (*n* = 23). These included nine longitudinal cohort or prospective studies, one mixed-method research study, and one case-control study (Table 1). It should be noted that in all studies the experience of violence was related to a previous period and that sleep problems continued over time. Consequently, cross-sectional studies always had a retrospective approach, and prospective studies were generally organized as repeated cross-sectional studies. The distinction between the two types of epidemiological design was often blurred. For example, the study by Thurston et al. (2019), was described by the authors as “a prospective cohort study” although it was a cross-sectional study with retrospective reporting of violent experiences.

**Table 1:** Studies on workplace violence and sleep disorders.

**References**	**Country**	**Study design**	**N. cases**	Quality^*^	**Type of population**	**Violence**			**Sleep**			**Results**	**Prevalence data**
						**Type of violence**	**Length of recall period**	**WV measure**	**Type of sleep problem**	**Length of recall period**	**Sleep measure**		
Zahid et al., 1999	Kuwait	Cross-sectional	101	2/10	A&E doctors	Physical and verbal	n.s.	*Ad hoc*	Insomnia	n.s.	*Ad hoc*	Exposure was associated with insomnia and other symptoms	86% of the exposed doctors reported symptoms, including insomnia.
Vartia, 2001^§^	Finland	Cross-sectional	949	4/10	General	Bullying	12 mths	LIPT	Use of drugs	n.s.	*Ad hoc* 1 item	Exposure was associated with use of sleep-inducing drugs	Prevalence of bullying: 10%. Prevalence of use of sleep-inducing drugs among bullied: 13%
Atawneh et al., 2003	Kuwait	Cross-sectional	81	2/10	Hospital nurses	Physical and verbal	n.s.	*Ad hoc* questionnaire	sleeplessness	n.s.	*Ad hoc* 1 item.	Exposure was associated with sleeplessness	Prevalence of WV: 86%. Prevalence of sleeplessness: 73%.
Arthur et al., 2003	USA	Cross-sectional	1,131	3/10	Mental health providers	Physical and verbal	n.s.	*Ad hoc* questionnaire	Being unable to sleep	n.s.	*Ad hoc* questionnaire	Exposure was associated with insomnia	Prevalence of WV: 61%. Prevalence of sleeplessness 9.0%
Eriksen et al., 2008^§^	Norway	Prospective	4,471	5/9	Nurse aids	Physical and verbal	3 mths	QPSNORDIC	Sleep quality	3 mths	1 item from the BNSQ	Exposure to violence and threats was associated with poor sleep quality (OR = 1.19; 95% CI = 1.01–1.40).	Prevalence of WV, 41.08% Prevalence of “poor sleep” at baseline: 29.7%, at follow-up: 37.3%.
Niedhammer et al., 2009^§^	France	Cross-sectional	7,694	7/10	General	Bullying	12 mths	LIPT	Sleep disturbances	n.s.	*Ad hoc* 2 items question	Exposure was associated with sleep disturbances in men (aOR = 4.40; 95% CI = 3.35–5.78) and women (aOR = 3.83 95% CI = 3.12–4.70)	Prevalence of bullying, 9.91% Prevalence of bullied people with sleep disturbances: 4.62%.
Takaki et al., 2010	Japan	Cross sectional	2,500	5/10	General	Bullying	6 mths	NAQ	Sleep disturbances	1 mth	PSQI	WV plays a mediating role in the relationship between job strain and sleep disturbances	Prevalence of WV: 81.2% No prevalence data on sleep problems
Rodriguez-Munoz and Notelaers, 2011	Belgium	Cross-sectional	4,068	3/10	General	Bullying	6 mths	NAQ	Sleep quality	n.s.	*Ad hoc*	Exposure was associated with poor sleep quality	No prevalence data
Lallukka et al., 2011	Finland	Prospective	7,332	5/9	General	Bullying	n.s.	*Ad hoc* 2 items question	Sleep problems	n.s.	*Ad hoc* 4 items question	Exposure was associated with sleep problems (OR = 1.69; 95% CI = 1.30–2.20 in female) (OR = 3.17; 95% CI = 1.85–5.43 in male).	Prevalence of bullying at baseline: 22.85%. Prevalence of sleep problems at baseline 20.2%; at follow-up: 24.8%.
Bambi et al., 2013	Italy	Cross-sectional	444	5/10	A&E nurses	Bullying	n.s.	*Ad hoc* questionnaire	Sleep disturbances	n.s.	*Ad hoc* questionnaire	Exposure was associated with complaints including sleep disturbances	81.6% nurses were victims of lateral hostilities No prevalence data on sleep problems
Ziemska et al., 2013^§^	Poland	Cross-sectional	1,096	2/10	University workers	Bullying	n.s.	*Ad hoc* questionnaire	Sleep disorders	n.s.	*Ad hoc* questionnaire	Exposure was associated with sleep disorders (OR = 3.43; 95% CI = 2.30–5.13)	Prevalence of exposure to bullying: 19.34%. Prevalence of sleep disorders: 10.94%. Prevalence of exposed people with sleep disorders: 11%
Park et al., 2013	Korea	Cross-sectional	10,039	7/10	General	Physical, verbal, and sexual	12 mths	*Ad hoc* questionnaire	Sleep problems	n.s.	*Ad hoc* 1 item question	Exposure to physical (aOR = 1.98; 95% CI = 1.06–3.68) or sexual violence (aOR = 3.47; 95% CI = 1.77–6.81), was associated with sleep problems	Prevalence of WV 6.9% Prevalence of sleep problems 5.1% (95% CI 4.7–5.5%).
Slopen and Williams, 2014	USA	Cross-sectional	2,983	7/10	General	Verbal (discrimination).	n.s.	Perceived Racism Scale, adapted	Sleep duration and difficulties	4 wks	*Ad hoc* 3 items question.	Exposure to workplace harassment was associated with shorter sleep duration (B = −0.09) and sleep difficulties (B = 0.04)	No prevalence data
Tutenges et al., 2015	Denmark	Cross-sectional	151	2/10	Bouncers	Physical and verbal	n.s.	*Ad hoc* questionnaire	Trouble sleeping	12 mths	*Ad hoc* 1 item question	Exposure was associated with sleep problems	Prevalence of WV 96%. Prevalence of sleeping problems 50.4%
Miranda et al., 2014	USA	Prospective	344	5/9	Home Care	Physical assaults	3 mths	*Ad hoc* questionnaire	Pain interference with sleep	n.s.	*Ad hoc* 1 item question	Exposure was associated with pain interfering with sleep at baseline (aPR = 1.8; 95% CI = 1.2–2.6) and at 2-year follow-up (PR = 2.2; 95% CI = 1.5–3.0)	Prevalence of WV: 55% at baseline, 25% in all three periods (“persistent violence”). Pain interfering with sleep at baseline: 42% and in 3 surveys: 41%.
Kostev et al., 2014^§^	Germany	Case-control	2,625	5/9	General	Mthbbing	n.s.	*Ad hoc*	Sleep disorder (ICD-10)	12 mths	*Ad hoc*	Exposure was associated with sleep disorders (OR = 2.4, *p* <0.05)	Prevalence of sleep disorders: 13.3% in workers reporting mobbing, 5.1% in workers without mobbing.
Min et al., 2014	Korea	Cross-sectional	7,007	6/10	General	Verbal & sexual	12 mths	*Ad hoc* 1 item question	Sleeping problems	n.s.	*Ad hoc* 1 item question	Exposure was associated with sleeping problems (male: PR = 2.3; 95%; CI = 1.7–3.2; female: 3.0; 95% CI = 1.9–4.7).	Prevalence of WV 7.2%. Prevalence of sleep problems: 5.12% (male: 6%, female: 4.3%)
Ovayolu et al., 2014	Turkey	Cross-sectional	260	2/10	Hospital Nurses	Bullying	n.s.	*Ad hoc* questionaire	Sleep disorders	n.s.	*Ad hoc*	Exposure was associated with sleep disorders	Prevalence of workplace bullying: 3.1%. Prevalence of health or sleeping problems: 66.2%.
Hansen et al., 2014	Denmark	Prospective	2,919	5/9	General	Bullying	n.s.	*Ad hoc* questionaire	Sleep problems	3 monnths	KSQ	Exposure was associated with poor sleep quality (3.56; 95% CI = 1.09;11.59)	Prevalence of bullying 12.16% No prevalence data on sleep problems
Hanson et al., 2015	USA	Cross-sectional	1,214	5/10	Home Care	Physical, verbal, and sexual	12 mths	*Ad hoc* questionaire	General sleeping troubles	n.s.	1 item from COPSOQ	Exposure was associated with sleep problems	Prevalence of WV: 61.3%. No prevalence data on sleep problems
Magee et al., 2015	Australia	Cross-sectional	1,454	6/10	General	Bullying	6 mths	NAQ	Sleep quality	1 mth	PSQI	Dose-response relationship between the number of WV episodes and sleep quality.	Prevalence of frequent bullying: 8.4%. No prevalence data on sleep problems
Bonde et al., 2016^§^	Denmark	Prospective	7,502	4/9	General	Bullying	n.s.	*Ad hoc* 1 item question	Sleep quality	3 mths	KSQ	Bullying at baseline significantly predicted sleep disturbance (ORa = 1.29; 95% CI = 0.9–1.7)	Prevalence of bullying, baseline 7.4% Prevalence of sleep problems, baseline 10.3% Prevalence of sleep problems in bullied people 15.5%
Yoo et al., 2016^§^	Korea	Cross-sectional	25,138	3/10	General	Physical, verbal, and sexual	1 mth	*Ad hoc* questionaire	Sleep disturbances	12 mths	*Ad hoc* 1 item question	WV was associated with sleep disturbance (OR = 3.773; 95% CI = 3.058–4.655). Lateral violence was associated with sleep disturbances (OR = 5.688; 95% CI = 4.189–7.723)	Prevalence of WV 6.0% Prevalence of sleep disturbance: 2.4%
Nabe-Nielsen et al., 2016	Denmark	Prospective	7,650	5/9	General	Bullying, unwanted sexual attention	n.s.	*Ad hoc* questionaire	Disturbed sleep	3 mths	KSQ	Disturbed sleep mediated 12.8% (95% CI = 8.1–19.8) of the association between bullying and long-term sickness absence, and 8.5% (95% CI = −0.45 to 37.1) of the association between unwanted sexual attention and long-term sickness absence	Prevalence of bullying varies across studies (WHB, 2006 and 2008; PRISME, 2007 and 2009): from 5 to 10% and unwanted sexual attention from 1 to 4%. No prevalence data on sleep problems
Hansen et al., 2016	Denmark	Prospective	3,278	5/9	General	Bullying	n.s.	*Ad hoc* 1 item question	Sleep problems	3 mths	KSQ	Exposure at baseline was associated with early awakening (β = 0.06; 95% CI = 0.01–0.11) and lack of restful sleep (β = 0.07; 95% CI = 0.02–0.11) at follow-up.	Prevalence of bullying 9.20% No prevalence data on sleep problems
Pitney et al., 2016	USA	Mixed-methods.	567	6/10	Athletic trainers	Bullying	6 mths	NAQ	Sleep disturbances	n.s.	SSI	Exposure was associated with sleep disturbances	Prevalence of bullying: 7.8%. No prevalence data on sleep problems.
Vedaa et al., 2016	Norway	Prospective	799	4/9	Shift working nurses	Bullying	6 mths	NAQ	Sleep problems	1 mth	BIS	Exposure predicted increased symptoms of insomnia over time.	No prevalence data
Acquadro Maran et al., 2017	Italy	Cross-sectional	1,842	4/10	Hospital	Stalking	n.s.	Stalking Questionnaire	Sleep disorders	n.s.	*Ad hoc* 1 item question	Stalking was significantly associated with sleep disorder.	Prevalence of stalking 13.9%. Prevalence of sleep disorders among victims of stalking: 50.7%
Gluschkoff et al., 2017	Finland	Prospective	4,988	5/9	Teachers	Physical and verbal	n.s.	*Ad hoc* 1 item question	Sleep disturbances	4 wks	JSPS	Exposure was associated with an increased rate of sleep disorders (RR 1.32; 95% CI = 1.15–1.52).	Prevalence of WV: 33% No prevalence data on sleep problems
Sun et al., 2017	China	Cross-sectional	3,016	3/10	Hospital	Physical, verbal and sexual	n.s.	*Ad hoc* questionaire	Sleep quality	n.s.	*Ad hoc* 1 item question	Exposure was negatively correlated with sleep quality (*r* = −0.281, *p* <0.001)	Prevalence of WV: 83.4%. No prevalence data on sleep problems.
Pekurinen et al., 2017	Finland	Cross-sectional	5,228	5/10	Nurses	Physical and verbal	12 mths	*Ad hoc* 1 item question	Sleep disturbances	4 wks	JSPS	Psychiatric nurses who experienced WV were less likely to suffer from sleep disturbances compared to nurses working in medical, surgical and emergency settings (OR = 0.65, *p* = 0.007 and OR = 0.39, *p* <0.001).	Prevalence of exposure to aggression by patients: 41%. Prevalence of sleep disturbances: 49%.
Zhang et al., 2018	China	Cross-sectional	1,024	2/10	Nurses	Physical, verbal and sexual	12 mths	*Ad hoc* 1 item question	Sleep quality	n.s.	*Ad hoc* 1 item question	WV exposure was negatively associated with sleep quality (*r* = −0.194, *p* <0.01) Psychological stress was a mediator in the relationship between violence and sleep quality.	Prevalence of WV: 75.4%. No prevalence data on sleep problems.
Karhula et al., 2018	Finland	Cross-sectional	9,312	5/10	Nurses	Physical and verbal	12 mths	*Ad hoc* questions	Sleep difficulties	4 wks	*Ad hoc* 1 item question	Permanent night workers reported difficulties in falling asleep more often than day and shift workers, but reported difficulties in maintaining sleep less often than other colleagues	Prevalence of WV: 53.90 Prevalence of insufficient sleep: 24.18%
Thurston et al., 2019	USA	Cross-sectional	304	5/10	General, women	Sexual harassment and assault	Longlife	BTQ	Sleep quality	1 mth	PSQI	Exposure to Sexual Harassment was associated with poor sleep (aOR = 1.89; 95% CI = 1.05–3.42). Exposure to Sexual assault was associated with poor sleep (aOR = 2.15; 95% CI = 1.23–3.77)	Prevalence of sexual harassment: 19%. Prevalence of sexual assault: 22%. No prevalence data on sleep problems

*A&E, Accidents and Emergency; n.s., not specified; mths, months; wks, weeks; LIPT, Leymann Inventory of Psychological Terror; NAQ, Negative Acts Questionnaire; BTQ, Brief Trauma Questionnaire; QPSNORDIC, General Nordic questionnaire for psychological and social factors at work; JSPS, Jenkins Sleep Problems Scale; KSQ, Karolinska Sleep Questionnaire; BIS, Bergen Insomnia Scale; PSQI, Pittsburgh Sleep Questionnaire Index; BNSQ, Basic Nordic Sleep Questionnaire; SSI, Semi-structured interviews*.

(*) *: Newcastle-Ottawa Quality Assessment Form for Cohort studies score (maximum score = 9) and Newcastle-Ottawa Quality Assessment Scale for Case-Control/Cross-sectional studies score (maximum score = 10)*.

(§) *Selected for meta-analysis*.

According to our evaluation, the prospective studies had a moderate to low quality score (ranging from 4 to 5 on the 9-point NOS scale, and from 2 to 7 on the 10-point NOS-A scale). Overall, the 10 cohort or case-control studies had an average score of 4.8 on 9 points on the NOS scale, the other 24 studies an average score of 4.2 points out of 10 of the NOS-A scale.

The selected studies had been conducted in the USA (Cousins and Fernández, 2019), Finland (Massar et al., 2019), Denmark (Massar et al., 2019), Korea (Hudson et al., 2019), Norway (Garbarino et al., 2016c), China (Garbarino et al., 2016c), Italy (Garbarino et al., 2016c), Kuwait (Garbarino et al., 2016c), France, Japan, Belgium, Poland, Germany, Turkey, and Australia, and involved a total of 119,361 workers.

The risk factors taken into consideration were: workplace bullying (13 studies), bullying and unwanted sexual attention (2 studies), mobbing (1 study), physical and verbal violence (8 studies), physical, verbal, and sexual violence (5 studies). Other studies considered physical assaults, verbal discrimination, verbal and sexual harassment, stalking, sexual harassment, and assaults as the independent variable. The experience of violence was analyzed retrospectively, generally by means of questionnaires, with a reference period that varied from 1 to 12 months. Some studies failed to indicate the length of the recall period.

Most studies (21 studies) investigated a general type of working population or focused on health care workers and social services (14 studies), although a few studies considered other populations (bouncers, athletic trainers, teachers, university workers).

Outcomes were described as “sleep problems” in 6 studies; “sleep disturbances” or “disturbed sleep” in 7 studies; “interference with sleep due to pain” in 1 study; “poor sleep quality” in 8 studies; “sleep difficulties” or similar terms in 7 studies; “sleep disorders” in 4 studies (explicit reference was made to ICD-10 in only one study), and “use of sleep-inducing drugs and sedatives” in 1 study. The sleep problems referred to a period ranging from 4 weeks to 12 months prior to the survey.

### Finding 1: Relationship Between WV and Sleep Problems

The first report of an association between WV and insomnia was published by Zahid et al. (1999). This was followed by further sporadic reports on this relationship (Vartia, 2001; Arthur et al., 2003). A more recent short-time longitudinal study by Eriksen et al. (2008) included WV among predictors of poor sleep quality in Norwegian nursing assistants and estimated a small increase in the risk (OR = 1.19; 95% CI = 1.01–1.40), whereas the retrospective French study conducted by Niedhammer et al. (2009) suggested that WV exposure could quadruple the risk of sleep problems (SPs).

Studies on violence and SPs have often focused on health care workers who are frequently subjected to physical attacks and verbal aggression (Magnavita and Heponiemi, 2012). These two conditions (being physically assaulted or threatened by patients/visitors, or being mistreated by superiors and colleagues) do not have the same effect on health. The literature indicates that lateral violence, which is less frequent than physical aggression from patients or visitors, seems to result in more severe health-related outcomes (Reknes et al., 2017), especially in younger and inexperienced workers (Magnavita and Heponiemi, 2011). In our review, the harmful effect of violence perpetrated by colleagues was confirmed in a study conducted in five hospitals in the Italian Region of Tuscany. Lateral hostilities among emergency and critical care nurses have been associated with SPs in victims (Bambi et al., 2013), and the same phenomenon has been observed in Turkish hospital nurses (Ovayolu et al., 2014). Lateral violence was among the predictors of insomnia in a longitudinal study on Norwegian shift nurses (Vedaa et al., 2016) and in many studies that focused specifically on workplace bullying (Lallukka et al., 2011; Rodriguez-Munoz and Notelaers, 2011; Ziemska et al., 2013; Hansen et al., 2014, 2016; Kostev et al., 2014; Magee et al., 2015; Bonde et al., 2016; Pitney et al., 2016).

The association between WV and SPs in health care workers has been corroborated by studies carried out in various parts of the world. A cross-sectional study conducted across eight provinces in China demonstrated that exposure to WV significantly affected the sleep quality of employees. Distressed workers manifested the most apparent impairment (Zhang et al., 2018). Another study on Chinese doctors revealed that exposure to WV significantly affected sleep quality and self-reported level of health (Sun et al., 2017). In 50 Japanese companies, workers exposed to violence had a higher rate of SPs and depression than non-exposed employees (Takaki et al., 2010). Data from the 4th Korean Working Conditions Survey indicated that WV was a factor affecting SPs (OR = 3.773; 95% CI = 3.058–4.655). The same study found that SPs were reported more frequently when the perpetrator was a colleague or boss (OR = 5.688; 95% CI = 4.189–7.723) rather than a client (OR = 2.992; 95% CI = 2.301–3.890; Yoo et al., 2016).

Similar data have been obtained from studies conducted in Western countries. Female homecare workers in Oregon reported a high frequency of physical assaults significantly associated with SPs (Hanson et al., 2015). Discrimination, workplace harassment, and incivilities were associated with shorter sleep duration and sleep difficulties in black, Hispanic, and white American adults (Slopen and Williams, 2014). Workplace bullying was strongly associated with SPs in the French working population, with an increased adjusted odds ratio in both men (aOR = 4.40; 95% CI = 3.35–5.78) and women (aOR = 3.83; 95% CI = 3.12–4.70; Niedhammer et al., 2009). A Danish longitudinal study showed that bullied subjects reported more SPs than those who were neither bullied nor witnesses to bullying at baseline (Hansen et al., 2014). A further Danish study on public and private office employees observed that poor sleep is among the health correlates of bullying (Bonde et al., 2016). The PRISME cohort study conducted in Denmark found that workplace bullying at baseline was associated with awakening problems and lack of restful sleep at follow-up, but not with overall SPs and disturbed sleep (Hansen et al., 2016). Bullying at work was associated with sleep disorders in Polish university workers (Ziemska et al., 2013). A Finnish Public Sector survey indicated that exposure to WV was associated with an increase in disturbed sleep (RR = 1.32; 95% CI = 1.15–1.52) that persisted also after exposure (RR = 1.26; 95% CI = 1.07–1.48; Gluschkoff et al., 2017). The Helsinki Health Study revealed that workers exposed to bullying at baseline reported SPs at follow-up (Lallukka et al., 2011). Workplace sexual harassment and sexual assaults have been associated with SPs in many studies (Zahid et al., 1999; Park et al., 2013; Ziemska et al., 2013; Hanson et al., 2015; Nabe-Nielsen et al., 2016; Yoo et al., 2016). Type 4 violence (e.g., stalking in the workplace), perpetrated by people who have a relationship with the worker, but not with the workplace (Magnavita and Magnavita, 2007), can also heavily interfere with sleep. Continual aggression on the part of the stalker leads to both physical and emotional effects in victims, the most frequent of which are sleep disorders (Acquadro Maran et al., 2017).

### Finding 2: Factors Affecting the Association of WV With SP

Researchers have sought to identify the occupational factors that may mediate the association of WV with SPs. Due to its well-known interference with biorhythms, night shift work was the first factor to be taken into consideration. In this review, researchers hypothesize that permanent night work, or alternating shifts that also include night work, are more harmful to sleep than daytime work. However, studies from the Finnish Public Sector database demonstrated that permanent night workers manifest inconsistent differences in sleep quality compared to day and shift workers. Since a slightly longer average length of sleep, fewer problems in maintaining sleep, and more difficulties in falling asleep were observed in night workers, the authors concluded that the type of shift alone cannot explain the association between WV and SPs (Karhula et al., 2018).

The relationship between WV and SPs in health care workers is complex and undoubtedly involves many factors such as the type of work performed, the relationship with patients, the level of worker engagement, the organization of work, the level of social support, and staff cohesion, all of which greatly influence the resilience of workers (Magnavita and Fileni, 2012; Magnavita, 2017). Interestingly, our review included a Finnish study that demonstrates that psychiatric nurses exposed to violence maintain better psychiatric well-being and experience fewer SPs than non-psychiatric nurses with a similar exposure (Pekurinen et al., 2017). These complex associations between WV, SPs, and work organization are observed not only in health care employees, but also in all types of workers. The findings of the Korean Working Conditions Survey indicated that workers exposed to workplace injustice (e.g., discrimination, harassment, or violence) had an ~2- to 3-fold increased risk for SPs (Min et al., 2014). In these workers, the frequency of work-related SPs was 5.1%. WV and the threat of violence were significantly associated with SPs in another Korean study (Park et al., 2013). Other studies showed that psychological stress can act as a partial mediator in the relationship between violence and sleep quality (Zhang et al., 2018), while organizational justice may have a protective effect (Gluschkoff et al., 2017).

Our review indicated that SPs were not always the main outcome of studies, but merely one of the symptoms associated with violence or resulting from it. In most cases, researchers reported psychiatric disorders such as anxiety, depression or burnout, or other parameters, such as sick leave or musculoskeletal disorders as outcome variables. In more recent studies, SPs were reported as a collateral factor that plays a moderating role in the relationship between the main variables. For example, studies on absenteeism showed that disturbed sleep and awakening difficulties mediated the association between bullying and long-term sickness absence (Nabe-Nielsen et al., 2016). Studies on clinical nursing home workers in Massachusetts showed that both musculoskeletal pain (Miranda et al., 2011) and sleep disorders (Miranda et al., 2014) increased in assaulted workers.

### Finding 3: Prevalence of SPs in Workers Experiencing Violence

In the studies selected, different types of WV were considered to be a causal factor. Some studies took into consideration only type 2, type 3, or type 4 violence, while some considered only physical or moral violence, and others focused on sexual violence. This heterogeneity may explain why WV prevalence ranges so widely from 3 to 96% in these studies. It follows that the overall prevalence of SPs in exposed workers also falls within a very large range extending from less than 5% (Yoo et al., 2016) to nearly 50% (Tutenges et al., 2015; Pekurinen et al., 2017). Although all the authors reported a high prevalence of SPs in workers exposed to violence, a number of researchers failed to make a comparison with non-exposed workers.

Some difficulty was encountered in comparing prevalence rates on account of the diversity of methods used to measure SPs. Using an *ad hoc* questionnaire, Atawneh et al. (2003) calculated the prevalence of insomnia in hospital nurses exposed to WV to be 73%, while a different *ad hoc* questionnaire administered by Arthur et al. (2003) indicated a 9.0% rate among mental health workers. According to Niedhammer et al. (2009), bullied subjects had a 4.6% rate of SPs, while Ziemska et al. (2013) found the rate to be 11%, and Bonde et al. (2016) reported a rate of 15%. In a study conducted by Vartia (2001), 13% of bullied workers were taking sleep-inducing drugs, while Acquadro Maran et al. (2017) observed that more than 50% of subjects exposed to workplace stalking had sleep disorders.

### Finding 4: Meta-Analysis

Our meta-analysis included 7 studies that published the data needed to calculate odds ratios. Of these, the study of Bonde et al. (2016) presented a repeated cross-sectional design, with measurements at baseline and at 2 and 4 years. Since these three measurements were considered to be different studies, a total of 9 studies were obtained.

The quality of the studies included in the meta-analysis ranged from 2/10 (Ziemska et al., 2013) to 7/10 (Niedhammer et al., 2009). The average quality of prospective or case-control studies included in meta-analysis was 4.7 out of 9, that of the other studies 4 points out of 10. All studies were observational. Most cases involved a general population, although one study focused on nursing assistants and another regarded university employees. Bullying was the principal type of violence, although one case involved mobbing, another physical and verbal violence, and one further study concerned physical, verbal and sexual violence. The length of recall periods for WV varied: 1 month (one study), 3 months (one study), and 12 months (two studies). All the other studies failed to specify the recall period. The length of recall periods for SPs was 3 months (two studies) or 12 months (two studies), while other studies failed to specify these data.

**Figure 2:**
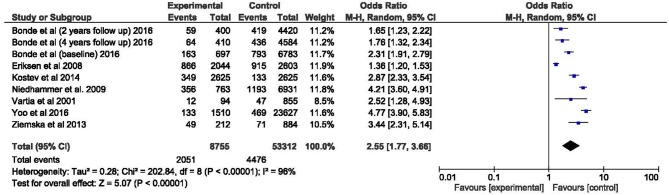
Meta-analysis of the association of workplace violence and sleep problems. Forest plot.

The overall odds ratio of WV and SPs was 2.55 (95% CI: 1.77–3.66). A highly significant overall level of significance (*p* < 0.001) was found for the meta-analysis, and a high degree of heterogeneity (*I*^2^ = 96%, *p* < 0.001) was observed among the studies selected (Figure 2).

As usual in the workplace, no study was a randomized trial. A single study (Yoo et al., 2016) alone included about half of the observed sample. Since this was also the study that reported the lowest level of quality, this certainly reduces the reliability of the estimate. However, it is easy to see that all studies lead to similar results and that confidence intervals are not too wide.

**Figure 3:**
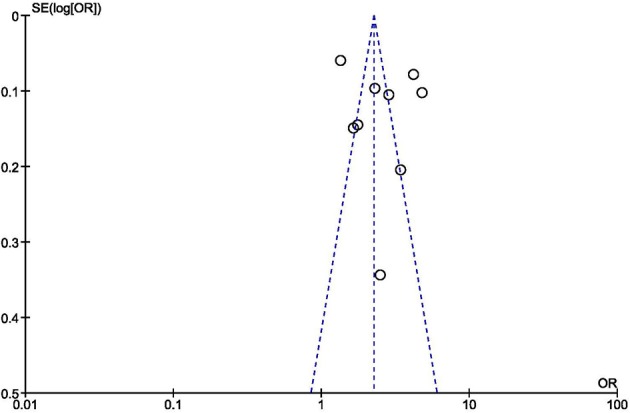
Meta-analysis of the association of workplace violence and sleep problems. Funnel plot.

The asymmetric funnel plot (Figure 3) indicated the presence of publication bias. In systematic reviews that select studies published in English, the exclusion of studies written in other languages leads to an obvious risk of bias. On the other hand, in reviews that include studies written in other languages, there is the risk of overestimating the contribution of the national literature. In this review, as only one study out of 34 was written in Italian, language bias can be considered negligible.

Overall, the studies agree that WV is associated with SPs, but the evidence of the strength of this association is low, due to inconsistency arising from methodological heterogeneity, low quality of studies and risk of bias.

## Discussion

The literature reviewed by the authors indicates, with low evidence, that WV is associated with an increased risk of SPs. All the studies included in this systematic review are concordant in reporting that workers exposed to violence manifest problems related both to the quantity of sleep (e.g., sleep loss) and the quality of sleep, i.e., difficulty in falling asleep, frequent interruptions, early awakenings, insomnia, nightmares. Different types of violence do not all result in the same degree of harmfulness. Verbal violence perpetrated by colleagues and superiors, known as lateral violence or bullying, appears to be the most harmful type. A consistently high prevalence of SPs in workers exposed to violence was found in the studies surveyed, and the risk for subjects who had experienced violence was significantly higher than for other workers. The meta-analysis yielded a pooled odds ratio of >2.5 for subjects exposed to violence compared to other workers, although this estimate is limited on account of the great heterogeneity of the studies.

Study disparities were mainly due to the type of violence investigated and the length of the recall period. The authors investigated different periods with consequent variations in the degree of accuracy, since increasing the recall period provides more information but increases the likelihood of recall error. Moreover, since WV was investigated in a retrospective way in all the studies, when interpreting the results, the authors often attributed a causal role to the experience of violence, in some cases without ascertaining whether the same SPs were present also in subjects who had not undergone violence. A considerable disparity was also present in the definition of SPs; in fact, only one research group provided the definition of “sleep disorder” as envisaged by ICD-10. All other researchers defined the outcome in different ways, using generic terms such as sleep disturbances, difficulties, troubles, problems, and poor quality. Only a few studies used standardized questionnaires to define violence or sleep, while most researchers developed *ad hoc* questionnaires, sometimes composed of a single question. The quality of studies, assessed by the NOS and NOS-A scales, was not sufficiently high. It is well-known that information contained in published articles does not always ensure a perfect evaluation of the quality of the work; indeed, it has been shown that referees tend to overestimate quality. A methodological review that compared the assessments of reviewers and authors demonstrated that the overall NOS score in the assessments made by reviewers was significantly higher than those made by authors (Lo et al., 2014). This review found that the overall quality of the studies selected was poor and that the latter together with the marked heterogeneity constituted the main limitations. Our review puts forward the idea that violence should be systematically identified in the workplace and that further research should study the effect of WV on sleep, since alterations in sleep may be a predictor of occupational impairment, with relevant consequences on workers' safety and health.

Our findings confirm and extend what has been shown in previous review studies. A review of the research conducted in Latin American countries underlined the association between WV and SPs, without being able to quantify the association, since the studies were descriptive, with insufficient analytical nature (Ansoleaga et al., 2015). In 2013, the Swedish Council on Health Technology Assessment (SBU) concluded that people who experience bullying at work also have more sleep disturbances than those who are not subjected to such exposure at work (SBU, 2013). Our study has expanded this statement, indicating that non-bullying forms of violence are also associated with SPs. An interesting review of the literature aimed at identifying future SPs in workers exposed to various psychosocial risk factors has been conducted in 2015 within the framework of the SBU (Linton et al., 2015). The authors identified 24 studies, only two of which had studied prospectively the relationship between bullying and SPs. Authors concluded that many psychosocial work factors have an impact on SPs, and this might be utilized in the clinic as well as for planning work environments. We confirm this statement and are confident that future, well-designed studies, will help in elucidating the relationships between WV and SPs, clarifying the underlying mechanisms.

### Neurophysiological Mechanisms

The studies included in this systematic review do not address the issue of neurophysiological mechanisms activated by WV. However, these mechanisms can be hypothesized on the basis of current neurophysiological literature. WV could be considered and experienced as a stressful life event. The significant individual differences in the consequences of stress exposure highlight the moderating influence of endogenous psychophysiological vulnerabilities (Rosenthal, 1970). Epigenetic regulation is essential for neural and brain functioning, and putative epi-mutations may play a role in the etiopathogenesis of many sleep and psychiatric diseases (Agorastos et al., 2019). Maintenance of DNA methylation and histone modifications is crucial for normal neurodevelopment and functioning of the brain (Ptak and Petronis, 2010). The stress response is highly complex, as can clearly be seen by the fact that identical stressors of equal strength elicit different reactions in different individuals. It is well-known that a stressful event (cognitive, physiological, etc.) can disrupt the sleep system, due to what has been described as sleep reactivity (Drake et al., 2014). Sleep reactivity plays a key aetiological role in vulnerability to insomnia and possibly other SPs. Physiologically and cognitive-emotionally induced hyper-arousal can interfere with sleep in a subset of the population (Kalmbach et al., 2018b) that is prone to experiencing excessively strong sleep responses to a wide range of environmental (workplace and work organization) and psychosocial stressors such as WV. Responses to WV that are related to sleep reactivity may be neurobiologically supported in autonomic dysregulation with parasympathetic activity as a potential autonomic marker of SPs. Persistent symptoms of hyper-arousal (not present before WV) include difficulty falling or staying asleep, irritability or outbursts of anger, difficulty concentrating, hypervigilance, and extreme startle response (Kalmbach et al., 2018a). Mood and anxiety disorders commonly co-occur with SP, resulting in more severe clinical impairment than does either disorder alone. Indeed, SP is a core feature of both depression and anxiety disorders (Motomura et al., 2013; Goldstein-Piekarski et al., 2019).

The basic mechanisms that link violence-related stress, SP, and neuro-psychiatric mechanisms are not fully elucidated. The default mode circuit (DMN), including the anterior medial prefrontal cortex (PFC), the posterior cingulate cortex, the angular gyrus (Greicius et al., 2009), and the negative affective circuit (NA), including amygdala, hippocampus, insula, and the dorsal and ventral portions of the PFC (Robinson et al., 2014) may play a role. DMN is genetically heritable and is observed when the brain is probed under task-free conditions, and typically when participants are instructed to reflect on their own spontaneously generated thoughts. Connectivity between nodes of the DMN and other networks implicated in mood and anxiety disorders fluctuate as a function of sleep stage. Profiles of DMN connectivity have been associated with sleep debt, fatigue, and tiredness, core features of anxiety and depression. Hypo-connectivity of the DMN and poor sleep quality are both relevant for mood and anxiety. The most compelling findings with respect to insomnia are a relative lack of deactivation of the DMN when compared to healthy controls, hyper-reactivity of the DMN in response to sleep-related stimuli, and hyper-connectivity of the DMN under task-free conditions. It has been hypothesized that the lack of disengagement may be reflective of increased self-reflective thought, rumination, and worry that may keep individuals from falling asleep. Mirroring the DMN profiles seen in insomnia, depression has been associated with over-activation and hyper-connectivity within the DMN.

The dorsal prefrontal sub-circuit has been preferentially implicated in appraisal and expression of emotion, and may be considered an “aversive amplification” sub-network of the negative affective circuits that serve to boost the processing of signals of potential threat. Complementing this function, the ventral sub-circuit has been implicated in automatic regulation of negative emotion. There is a striking overlap between the pattern of NA network dysfunction, SP, and anxiety and depression. Indeed, both hyper-reactivity of the amygdala and hypo-connectivity of medial prefrontal cortex-amygdala are theorized to contribute to the development and maintenance of emotional distress that underlies the maladaptive behaviors commonly observed in anxiety disorders, including increased negative bias, increased threat responsivity, and poor emotional regulation. Moreover, the degree of reactivity and regulation in these nodes correlates with subjective experiences of emotional distress as well as emotional behaviors (Goldstein-Piekarski et al., 2019).

**Figure 4:**
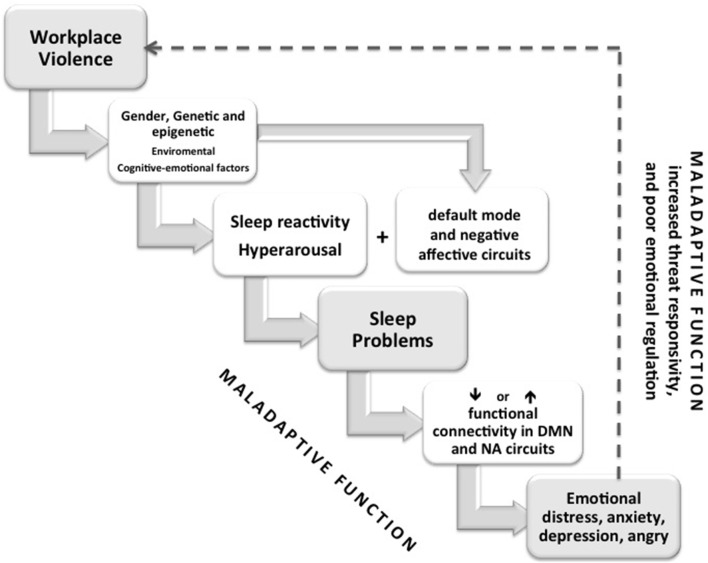
Conceptual diagram depicting the effect of WV stress on sleep problems across individual features (gender, genetic, environmental) with sleep reactivity, hyperarousal, and the alterations of two brain circuits genetically heritable (default mode circuit—DMN and the negative affective circuits—NA). Connectivity between nodes of the DMN, NA and other networks implicated in mood and anxiety disorders fluctuate as a function of sleep stage. Different sleep problems could induce hyper-reactivity or hypo-connectivity of different nodes of circuits manly mPFC-amygdala that, in turn, contribute to the development and maintenance of emotional distress that underlies the maladaptive behaviors commonly observed in anxiety disorders, including increased negative bias, increased threat responsivity, and poor emotional regulation. Moreover, subjects with sleep problems, with a genetic predisposition and an epigenetic modification could be involved in hostile and aggressive behavior (maladaptive behaviors) at workplace for a loss of emotional control and regulation of aggression (dashed line).

SP has been shown experimentally to induce negative mood states, as well as a negative affective network profile mimicking that of anxiety and depression. The improvement of sleep quantity/quality may normalize these brain changes (Goldstein-Piekarski et al., 2019). These findings suggest that at least for some individuals, maladaptive negative affective network function may be an intermediate step between sleep disruption and anxiety and mood features, and that sleep is a modifiable target through which emotional distress can be reduced (Figure 4).

Long work hours, night-owl lifestyles, and extensive shift work are major contributors to sleep loss and a consequent increase in the risk for anxiety and depression (Abad and Guilleminault, 2005). Cheung et al. performed a cross-sectional observational study showing how acute occupational sleep deprivation and a frequently disrupted sleep cycle induce DNA damage and changes in antioxidant capacity (Cheung et al., 2019). These observations are concordant with findings of genotoxicity in sleep-deprived animals and elderly adults (Tenorio et al., 2013).

Therefore, ensuring an adequate amount of sleep is an important lifestyle factor that should be given greater attention to the management of mental health and WV. Sleep deprivation weakens the ability to inhibit aggression and enhances impulsivity (Kahn-Greene et al., 2006) with delay discounting, risk-taking, sensation-seeking, and a lack of behavioral response inhibition to negative emotional circumstances. The latter mediates the relationship between SPs and unwanted or context-inappropriate aggressive responses. Sleep restriction or deprivation mainly affects sleep-wake rhythms in shift and night workers, but it may also often alter or disrupt daily rhythms of activity, light exposure, eating patterns, drinking, body temperature, physiological activity, smoking, and sleep-wake dependent neural and endocrine variables (Garbarino et al., 2002; Kecklund and Axelsson, 2016). These neuropathological mechanisms can partially explain a number of extreme conditions, but they do not appear to adequately interpret the long-term mechanisms by which low-grade WV alters sleep.

Almost all the studies included in our review have evaluated the potential effect of violence on both the quantity and the quality of sleep. However, very few attempts have been made to ascertain whether an inverse causality also exists between these two variables, namely that the presence of SPs promotes WV. In point of fact, violence at work can be considered a stress factor, and it is well-known that there is a close relationship between violence and stress (Chirico, 2015, 2017). Recent longitudinal studies have shown that violence and stress are in a cyclic relationship, i.e., violence increases stress in the worker, and the distressed worker is prone to violence (Magnavita, 2013a, 2014). This topic was addressed in the German Workplace Bullying and Harassment longitudinal study in which the authors evaluated the occurrence of SPs after exposure to different workplace stressors, and the reverse causation concluded that SPs might also prospectively predict subjective role stressors (Hansen et al., 2018). In this context, it is interesting to note that a case-control study reported a higher prevalence of diseases in general, and SPs in workers who had experienced mobbing (Kostev et al., 2014). However, further studies are needed to ascertain whether an inverse relationship exists between SPs and WV.

In recent years, our society has witnessed an increase in the productive and social conditions that interfere with sleep (Fischer et al., 2014). Workers are presumed to be the major victims of sleep deprivation epidemics and the 24/7 society, even if time-use studies have not always corroborated this hypothesis (Lamote de Grignon Pérez et al., 2019). Aging of the population and the workforce has also exposed older people who often suffer from sleep disorders or have difficulty adapting their biorhythms to occupational risks (violence included; Flower et al., 2019). Furthermore, since the continual increase in violence in the workplace has recently been described as an “epidemic” (Rousseau et al., 2019), this study emphasizes the need to prevent violence in the workplace and improve sleep hygiene in workers.

Only a few countries in the world have included violence among the risks that must be prevented in the workplace and have consequently introduced appropriate health and safety regulations (Chirico et al., 2019). The quantity and quality of sleep are often not included among the occupational parameters to be monitored, and no widespread systems have been introduced for managing the risk of sleepiness at work (Costa et al., 2013; Magnavita, 2013b).

### Strengths and Limitations of This Study

A limitation of this systematic review and meta-analysis of the literature on the relationship between occupational violence and sleep problems is due to the heterogeneous nature of the selected studies that are not always of the highest quality. The principal strength of our research lies in the fact that it is the first attempt to systematize the relationships between WV and SPs. The secondary objective of our study, which was to analyse the neurophysiological mechanisms underlying the association found, could only be carried out in part, because the studies surveyed did not directly address the topic. The current limitations of our knowledge on this relationship should encourage researchers to conduct further higher quality studies.

## Conclusions

In conclusion, the association observed between common forms of workplace violence and changes in the sleep of a significant number of workers suggests that a concerted effort should be made to address all types of WV and to identify early alterations in workers' sleep patterns before these lead to harmful effects.

Researchers, physicians and managers share responsibility for promoting these efforts, even in countries where occupational safety and health standards do not compel the employer to prevent WV. Sleep health promotion campaigns should be introduced in conjunction with environmental, organizational and individual measures to prevent violence in the workplace.

The importance of this study lies above all in stressing that WV and SPs in workers are strongly connected. Subsequent studies may clarify to what extent particular forms of violence, for example, bullying or physical aggression, are correlated with sleep and therefore with safety and health in the workplace. Meanwhile, managers, physicians, and all stakeholders should engage in preventive measures to adequately control the phenomenon.

## Data Availability

The raw data supporting the conclusions of this manuscript will be made available by the authors, without undue reservation, to any qualified researcher.

## Author Contributions

FC selected the studies and drafted the manuscript. NM selected the studies and revised the manuscript. IC and EL extracted data. SG evaluated the neurophysiologic data. ED performed statistical analyses.

### Conflict of Interest Statement

The authors declare that the research was conducted in the absence of any commercial or financial relationships that could be construed as a potential conflict of interest.
